# Real-world analysis of ruxolitinib in myelofibrosis: interim results focusing on patients who were naïve to JAK inhibitor therapy treated within the JAKoMo non-interventional, phase IV trial

**DOI:** 10.1007/s00277-023-05458-1

**Published:** 2023-10-04

**Authors:** Steffen Koschmieder, Susanne Isfort, Clemens Schulte, Lutz Jacobasch, Thomas Geer, Marcel Reiser, Michael Koenigsmann, Bernhard Heinrich, Jürgen Wehmeyer, Eyck von der Heyde, Hans Tesch, Benedikt Gröschl, Petra Bachhuber, Susanne Großer, Heike L. Pahl

**Affiliations:** 1 Department of Hematology, Oncology, Hemostaseology, and Stem Cell Transplantation, Faculty of Medicine, RWTH Aachen University, Aachen, Germany; 2 Center for Integrated Oncology, Aachen Bonn Cologne Düsseldorf (CIO ABCD), Aachen, Germany; 3 Gemeinschaftspraxis Für Hämatologie Und Onkologie, Dortmund, Germany; 4 Gemeinschaftspraxis Hämatologie - Onkologie, Dresden, Germany; 5 Medizinische Klinik III, Diakonie-Klinikum Schwäbisch Hall, Schwäbisch Hall, Germany; 6 Praxis Internistischer Onkologie Und Hämatologie, Cologne, Germany; 7 Onkologisches Ambulanzzentrum (OAZ) Hannover, Hannover, Germany; 8 Hämatologisch-Onkologische Praxis Heinrich/Bangerter, Augsburg, Germany; 9 Gemeinschaftspraxis Für Hämatologie Und Onkologie, Münster, Germany; 10 Onkologische Schwerpunktpraxis Dres. Ingo Zander und Eyck von der Heyde, Hannover, Germany; 11 Onkologische Gemeinschaftspraxis am Bethanien-Krankenhaus, Frankfurt/Main, Germany; 12 Providing services for Novartis Pharma GmbH, Nürnberg, Germany; 13 Novartis Pharma GmbH, Nürnberg, Germany; 14 Department of Medicine I, Medical Center – University of Freiburg, Faculty of Medicine, University of Freiburg, Freiburg, Germany

**Keywords:** JAKoMo clinical trial, Myelofibrosis, JAK1/2 inhibitors, Ruxolitinib, Real-world evidence, Patient-reported outcome measures (PROMs)

## Abstract

Ruxolitinib (RUX) is a Janus kinase 1/2 inhibitor (JAKi) approved in the EU for treating disease‑related splenomegaly or symptoms in adults patients with myelofibrosis (MF). This is an interim analysis of JAKoMo, a prospective, non‑interventional, phase IV study in MF. Between 2012–2019 (cutoff March 2021), 928 patients (JAKi-naïve and -pretreated) enrolled from 122 German centers. This analysis focuses on JAKi-naïve patients. RUX was administered according to the Summary of Product Characteristics. Compared to the COMFORT-I, -II, and JUMP trials, patients in JAKoMo were older (median 73 years), had poorer Eastern Cooperative Oncology Group (ECOG) performance statuses (16.5% had ECOG ≥ 2), and were more transfusion dependent (48.5%). JAKoMo represents the more challenging patients with MF encountered outside of interventional studies. However, patients with low-risk International Prognostic Scoring System (IPSS) scores or without palpable splenomegaly were also included. Following RUX treatment, 82.5% of patients experienced rapid (≤ 1 month), significant decreases in palpable spleen size, which remained durable for 24 months (60% patients). Symptom assessment scores improved significantly in Month 1 (median –5.2) up to Month 12 (–6.2). Common adverse events (AEs) were anemia (31.2%) and thrombocytopenia (28.6%). At cutoff, 54.3% of patients had terminated the study due to, death, AEs, or deterioration of health. No new safety signals were observed. Interim analysis of the JAKoMo study confirms RUX safety and efficacy in a representative cohort of real-world, elderly, JAKi-naïve patients with MF. Risk scores were used in less than half of the patients to initiate RUX treatment.

Trial registration: NCT05044026; September 14, 2021.

## Background

Myelofibrosis (MF) is a chronic, *BCR*::*ABL*–negative myeloproliferative neoplasm (MPN) that is characterized by changes in blood counts, splenomegaly, disease-associated symptoms, and bone marrow (BM) fibrosis. It is caused by constitutive activation of the Janus kinase (JAK)/signal transducer and activator of transcription (STAT) pathway [1] through acquisition of one of the so called “driver mutations”: the *JAK2V617F* point mutation [2–5] or mutations in the genes encoding calreticulin (*CALR*) [6, 7] or the thrombopoietin receptor (*MPL*) [8]. MF may be primary (PMF) or evolve from polycythemia vera (PV) or essential thrombocythemia (ET) as a secondary MF (post–PV-MF [PPV-MF] or post–ET-MF [PET-MF], respectively). PMF can present as prefibrotic MF or as overt MF [9].

The small-molecule JAK1 and JAK2 inhibitor (JAKi) ruxolitinib (RUX) was a first-in-class drug and has significantly altered the treatment of MF since its approval in 2011/2012. Prior to this, only hydroxyurea and older drugs such as pipobroman or busulfan had been available for patients (patients) who were not eligible for allogeneic transplantation, and prognosis of high-risk patients was poor.

RUX was approved in the United States (U.S.) in 2011 and in the European Union (EU) in 2012 following the results of two pivotal, randomized, phase III trials (the COMFORT-I and -II trials). In both studies, patients receiving RUX exhibited significant reductions in spleen volume and symptom burden [10, 11]. Whereas the individual trials were not powered to demonstrate survival benefits, a pooled analysis of both trials showed a survival benefit of patients treated with RUX [12]. In the U.S., RUX is approved for patients with intermediate-1 (int-1), intermediate-2 (int-2), and high-risk myelofibrosis. In contrast, in the EU, approval has been granted independent of clinical risk scoring, and RUX is indicated for patients with MF-associated splenomegaly and/or disease-related symptoms.

A global expanded access clinical trial, termed JUMP, was conducted to provide expanded access to RUX following approval until the drug became fully available. The final results of this trial have been published [13]. In this analysis, data from more than 2,000 RUX-treated patients with MF were reported, including patients with low platelet counts (< 100 × 10^9^/L) and patients without splenomegaly, both of whom had not been included in the COMFORT trials. RUX led to clinically meaningful reductions in palpable spleen length and MF-associated symptoms, even in patients with low platelet counts. Symptom improvements were also seen in patients without splenomegaly [13]. However, to be eligible for the JUMP trial, patients needed to meet inclusion/exclusion criteria (Table 1), and, therefore, this study population did not represent the current “real-world” scenario of MF patients receiving RUX.

**Table 1:** Comparison of main inclusion and exclusion criteria from the JAKoMo, JUMP, and both COMFORT trials [10–13]

Parameter	JAKoMo	JUMP	COMFORT-I	COMFORT-II
Diagnosis of PMF, PET-MF, or PPV-MF according to WHO 2008	√	√	√	√
ECOG	No requirements	0–2	0–3	0–3
Risk assessment	Independent of IPSS	IPSS high + intermediate-2 + intermediate-1 with splenomegaly ≥ 5 cm BCM	IPSS high and intermediate-2	IPSS high and intermediate-2
Peripheral blast count	No requirements	< 10%	< 10%	< 10%
Lab assessments	No requirements	Direct bilirubin < 2 ULN; ALT < 2.5 ULN; coagulation parameters < 1.5 ULN (PT, PTT, INR)	Direct bilirubin < 2 ULN; ALT < 2.5 ULN; creatinine ≤ 2.0 mg/dl	Direct bilirubin < 2 ULN; ALT < 2.5 ULN; creatinine ≤ 2.0 mg/dl
Blood count	No requirements; according to label	ANC > 1000/µL; platelets ≥ 50.000	ANC > 1000/µL; platelets ≥ 100.000	ANC > 1000/µL; platelets ≥ 100.000
Splenomegaly at baseline	No requirements	≥ 5 cm BCM only in intermediate-1 patients	≥ 5 cm BCM	≥ 5 cm BCM
Previous treatment	No requirements; according to label	No requirements	Resistant or refractory to available treatment; intolerant, no candidates	Patients on treatment
Treatment indication	No requirements; according to SmPC label	No requirements	IPSS high risk or spleen ≥ 10 cm BCM or symptom burden (three in at least two items)	No requirements
Comorbidities	No requirements	Patients with paroxysmal atrial fibrillation excluded	Patients with paroxysmal atrial fibrillation excluded; RR systolic ≤ 160; RR diastolic ≤ 100;	Patients with paroxysmal atrial fibrillation excluded; RR systolic ≤ 160; RR diastolic ≤ 100;

Abbreviations: *ALT* alanine aminotransferase, *ANC* absolute neutrophil count, *INR* international normalized ratio,* PTT* partial thromboplastin time, *RR* respiratory rate, *ULN* upper limit of normal

The JAKoMo clinical trial (a prospective, two-arm, non-interventional study of RUX in patients with MF) was established in 2012 as a post-authorization, non-interventional, phase IV study and includes two different cohorts of patients: patients who were RUX naïve (Arm A) and those who were already receiving RUX (Arm B). Patients were included according to the drug label without any further restrictions or further inclusion/exclusion criteria. Hence, JAKoMo patients constitute a representative sampling of real-world patients receiving RUX. This is an interim analysis, focusing on RUX-naïve patients only (Arm A), with a median of 1.5 years of follow-up, reporting “real-world” data from RUX-treated MF patients.

## Methods

### Study design

JAKoMo is a two-arm, open-label, phase IV, non-interventional study of patients with MF who were either JAKi naïve or pretreated with JAKi. Patients ≥ 18 years with a diagnosis of PMF, according to the World Health Organization classification, or PPV-MF or PET-MF, according to the International Working Group for Myeloproliferative Neoplasms Research and Treatment criteria [14, 15], who were suitable for in-label treatment with RUX were eligible. Between September 2012 and September 2019, 928 patients (Arm A: *n* = 464 JAKi-naïve patients; Arm B: *n* = 464 JAKi-pretreated patients) eligible for analysis were enrolled across 122 centers in Germany. The current interim analysis, with a data cut-off of March 2021, focuses on the 464 JAKi-naïve patients (Arm A only). RUX was administered according to the Summary of Product Characteristics (SmPC). Drug dose and utilization, safety, and tolerability, as well as efficacy, including pt-reported outcomes, were documented. Pt-reported outcomes were assessed using the Myeloproliferative Neoplasm-Symptom Assessment Form (MPN-SAF) total symptom score (TSS), which has a possible range of 0–100, with 100 representing the highest level of symptom severity [16].

Starting doses of RUX were based on platelet counts according to the SmPC. Dose reductions or interruptions were also recommended according to the SmPC. Patients were observed for up to 36 months after enrollment, unless discontinuation criteria according to the SmPC were met.

Adverse events (AEs) and concomitant diseases were coded according to the Medical Dictionary for Regulatory Activities (MedDRA) version 23.1.

The study was sponsored by Novartis Pharma GmbH (Novartis) and designed by Novartis in collaboration with S. Koschmieder as the medical leading investigator. The study was approved by the institutional review boards of the respective institutions (leading ethics committee: Ethics Committee at the Faculty of Medicine, RWTH Aachen University, Aachen, Germany) before enrollment of patients and was conducted in accordance with the principles of the Declaration of Helsinki. The trial is registered with ClinicalTrials.gov (NCT05044026).

All patients provided written informed consent.

### Statistics

The study analysis used epidemiologic methods with primary use of descriptive statistical methods. All data were analyzed descriptively. In cases of confidence intervals or *p* values, the analyses were also descriptive; therefore, no alpha adjustment for multiple tests was performed.

The analysis was performed on the full analysis set population, which included patients with informed consent; a diagnosis of PMF, PPV-MF, or PET-MF; and documented administration of RUX during the study. Pt cohorts were separated into JAKi-naïve patients (Arm A) and JAKi-pretreated patients (Arm B).

Concomitant medications were analyzed using the Anatomical Therapeutic Chemical classification system (Level 1) preferred terms (Patients) according to the drug dictionary of the World Health Organization.

AEs were coded using MedDRA version 23.1 and were analyzed using frequency tables, presenting the numbers and percentages of subjects having any AE, having an AE in each primary system organ class, and having each individual AE (PT).

## Results

### Comparison to previously published, similar cohorts

The JAKoMo cohort is the first large, “real-world” cohort to be reported following RUX approval in the EU. It comprises a broader range of patients than enrolled in the COMFORT and JUMP trials. Table 1 compares the inclusion and exclusion criteria of the COMFORT-I/-II and JUMP trials to JAKoMo. Pt characteristics, shown in Table 1, were markedly different between the JAKoMo cohort and the three previous cohorts. Notably, only a subgroup of the JAKoMo trial population could have joined one of the other three trials, even though the patients in JAKoMo were treated within the indications listed in the SmPC.

Patients in the JAKoMo trial were enrolled mainly through office-based hematologists/oncologists (88% of patients), much fewer at community hospitals (9% of patients), and a minority at academic centers (2% of patients). Individual centers enrolled between one and 27 patients. Mean study duration of all patients was 20.2 months, with similar durations among patients with PMF, PPV-MF, and PET-MF. At the time of analysis, 54.3% of all patients had dropped out of the study, the major reasons being death, AEs, and deterioration of general health. 79 patients suffered from an adverse event which led to treatment termination. Main system organ classes for AEs leading to treatment discontinuation and drop out of the study were: general disorders and administration site conditions (18 patients), blood and lymphatic system disorders (17 patients with e.g. thrombocytopenia in 9 patients), gastrointestinal disorders (12 patients), and neoplasms (benign, malignant and unspecified) (17 patients). Allogenic stem cell transplantation was performed in 5 patients. The present analysis focuses exclusively on the JAKi-naïve patients (Arm A).

### Patients/demographics

Median age was 73 years and, thus, remarkably higher than in the COMFORT-II trial, and 53% of patients were male, a slightly lower percentage than in COMFORT-II. Median body mass index (BMI) was 24.3. Around 9% and 14% of patients were active or previous smokers, respectively. Patients who dropped out did not significantly differ from patients who had completed the study regarding sex or BMI; however, study completers tended to be younger (70 years vs 74 years) and non-smokers (4.5% vs 10%). For detailed pt demographics, please see Table 2.

**Table 2:** General characteristics of patients in arm A (*n* = 464) (no prior JAKi treatment)

Patient characteristics			Number of patients with available data
Gender, n (%)	Male	244 (52.6)	464
	Female	220 (47.4)	
Median age, years		73 (range 32–95)	464
MPN subtype, n (%)	PMF	310 (66.8)	464
	PET-MF	55 (11.9)	
	PPV-MF	96 (20.7)	
	No diagnosis	3 (0.6)	
Median time since first confirmed diagnosis, months		24.5	
IPSS (documented by investigator)	Low risk	23 of 194 (11.9%)	194
	Intermediate-1 risk	47 of 194 (24.2%)	
	Intermediate-2 risk	79 of 194 (40.7%)	
	High risk	45 of 194 (23.2%)	
	Missing data	270 (58.2%)	
Median BMI		24.3 (range 16.0–45.0)	464
Baseline ECOG	0	132 (29.9%)	
	1	236 (53.5%)	
	2	64 (14.5%)	
	3	9 (2.0%)	
	4 or 5	0	
	Missing data	23 (4.9%)	
Palpable spleen at enrollment, n (%)	Yes	240 (83)	289
	No	49 (17)	
Median baseline blood count	Leukocytes	13.3 × 10^9^/L	464
	Hemoglobin	11.1 g/dL	
	Platelets	287 × 10^9^/L	
Need for blood transfusion at baseline, n (%)	Strong need for transfusion (> 4 RBC units/month)	15 (3.2)	464
	Moderate need for transfusion (2–4 RBC units/month)	49 (10.6)	
	Low need for transfusion (< 2 RBC units/month)	161 (34.7)	
	No need for transfusion	239 (51.5)	
Molecular assessment in the last year before baseline, n (%)	*JAK2V617F* mutation	110 (69.2)	151 (32.5%)
	*MPL* mutation	3 (1.9)	
	*Calreticulin* mutation	3 (1.9)	
Smoking status, n (%)	Non-smoker	340 (77.1)	441
	Ex-smoker	63 (14.3)	
	Smoker	38 (8.6)	

Abbreviations: *BMI* body mass index, *ECOG* Eastern Cooperative Oncology Group, *IPSS* International Prognostic Scoring System, *JAK* Janus kinase, *RBC* red blood cell, *MPN* myeloproliferative neoplasm, *PET-MF* post-essential thrombocythemia myelofibrosis, *PMF* primary myelofibrosis, *PPV-MF* post polycythemia vera myelofibrosis, *MPL* thrombopoietin receptor

### IPSS risk groups and ECOG performance status

International Prognostic Scoring System (IPSS) groups were remarkably different from the COMFORT-II trial. Altogether, 12%, 24%, 41%, and 23% of patients were judged by their investigators to belong to the low-risk, int-1–risk, int-2–risk, and high-risk groups, respectively; however, there was a large fraction of patients (270/464 = 58%) not assigned to any IPSS group by an investigator. When we calculated the IPSS using the individual data provided in the electronic case report form (which was only possible for 108/464 patients, due to missing data), the fraction of low-risk to int-1–risk patients was confirmed to be around 39%, thus differing remarkably from the COMFORT-II trial, which only included int-2–risk and high-risk patients. Despite their lower risk scores, our cohort contained fewer patients not limited by their Eastern Cooperative Oncology Group (ECOG) performance status (ECOG 0) and a higher proportion of patients with an ECOG performance status 2–3 compared with COMFORT-II. Because of our permissive inclusion criteria, the JAKoMo cohort unbiasedly reflects the current population of MF patients receiving RUX in Germany.

### Time to enrollment and symptoms at diagnosis/enrollment

**Fig. 1:**
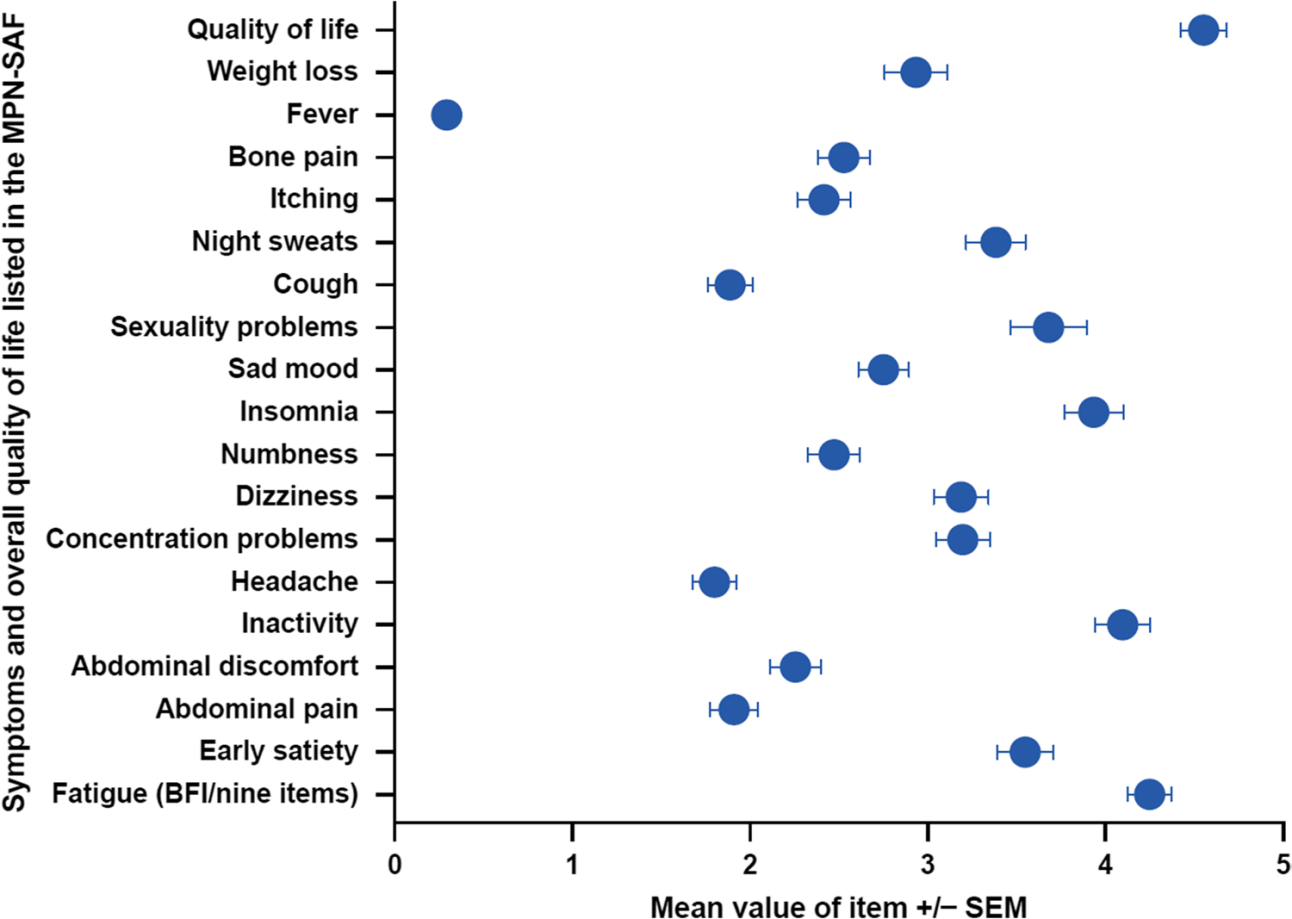
MPN-SAF questionnaire results at baseline. Mean with SEM (*n* = 464). Symptom severity was rated on a scale from 0 (absent/as good as it can be) to 10 (worst imaginable/as bad as it can be). MPN-SAF TSS has a possible range of 0–100, with 100 representing the highest level of symptom severity. Abbreviations: *BFI* Brief Fatigue Inventory, *SEM* standard error of the mean, *MPN-SAF* Myeloproliferative Neoplasm Symptom Assessment Form, *TSS* total symptom score

Median time from confirmed diagnosis to enrollment was 24.5 months, with PMF patients being enrolled into the trial after a shorter interval than patients with PPV-MF or PET-MF. The most common symptoms listed by physicians as reasons for suspecting MF were abnormal lab results and an enlarged spleen, followed by general weakness and formal constitutional symptoms. At trial enrollment, most patients suffered from fatigue, inactivity, and insomnia (Fig. 1).

### Concomitant medications and comorbidities

Patients had a median number of three comorbidities when entering the trial (Table 3). These pre-existing comorbidities included vascular disorders (48.9% of patients), with arterial hypertension being the most frequent. Interestingly, significantly fewer patients with PET-MF had pre-existing vascular disorders (30.9% of patients) than patients with PMF (50.3% of patients) or PPV-MF (56.3% of patients). Metabolism and nutrition disorders such as hyperuricemia and diabetes mellitus were present in 31% of patients, with only slight differences in frequencies between the three MF subentities. Overall, pre-existing cardiac disorders were present in 23.3% of patients, with a higher incidence in patients with PPV-MF compared with patients with PMF or PET-MF. Notably, pre-existing infections were reported in 6.5% of patients, with only two cases (0.4% each) of hepatitis B and zoster and only one case of previous tuberculosis. Also, skin ulcers were rarely reported (around 1% of patients).

**Table 3:** Comorbidities at time of and/or before entering the trial, coded according to MedDRA version 23.1 (*n* = 464)

System organ class	Preferred Term	Number of patients, *n* (%)
Vascular disorders		227 (48.9)
	Hypertension	206 (44.4)
Metabolism and nutrition disorders		144 (31.0)
	Hyperuricemia	41 (8.8)
	Type 2 diabetes mellitus	30 (6.5)
Cardiac disorders		108 (23.3)
	Coronary artery disease	43 (9.3)
	Atrial fibrillation	31 (6.7)
Neoplasms (benign, malignant, and unspecified, including cysts and polyps)		72 (15.5)
Nervous system disorders		71 (15.3)
Blood and lymphatic system disorders		64 (13.8)
	Anemia	28 (6.0)
Musculoskeletal and connective tissue disorders		62 (13.4)
Surgical and medical procedures		61 (13.1)
Renal and urinary disorders		54 (11.6)
Respiratory, thoracic, and mediastinal disorders		53 (11.4)
Endocrine disorders		51 (11.0)
	Hypothyroidism	33 (7.1)
Gastrointestinal disorders		50 (10.8)
Psychiatric disorders		36 (7.8)
Hepatobiliary disorders		30 (6.5)
Infections and infestations		30 (6.5)
Reproductive system and breast disorders		25 (5.4)
Skin and subcutaneous tissue disorders		23 (5.0)

Importantly, pre-existing malignancies were found in 72 patients (15.5%); these were similarly distributed among PMF, PPV-MF, and PET-MF patients, with the most frequent malignancies being prostate and breast cancers.

The most frequent pre-existing cytoreductive medication was hydroxyurea (30.2%), followed by anagrelide (approximately 5%), while only six patients had received interferon. Deferasirox had been administered in 3.9% of patients.

### Baseline blood counts/spleen size/BM/molecular testing

Baseline blood counts can be found in Table 2. At baseline, 22.1% of patients had a leukocyte count exceeding 25 × 10^9^/L, 65.5% of patients had a slightly elevated leukocyte count (> 10 × 10^9^/L), and 6.2% of patients were leukocytopenic (white blood cell [WBC] count < 4 × 10^9^/L). Median hemoglobin level at baseline was 13.3 g/dL. Transfusion dependency was present in 48.5% of patients (for transfusion frequency, please see Table 2). Median platelet count at baseline was 287 × 10^9^/L, with 272 (66.0%) patients having a platelet count > 200 × 10^9^/L, 83 (20.1%) patients with a count between 100 and 200 × 10^9^/L, 51 (12.4%) patients with a count between 50 and 100 × 10^9^/L, and five (1.5%) patients with a count < 50 × 10^9^/L.

Splenomegaly was reported by physicians in 83% of patients; however, spleen palpation at baseline was only documented in 62.7% of patients. Median spleen length at baseline was 13.9 cm below the costal margin (BCM), with a median size of 17 cm BCM for PPV-MF patients. Median spleen length by computerized tomography (CT) or magnetic resonance imaging (MRI) scan was 19.5 cm.

In 87.5% (*n* = 406) of the patients, a bone marrow biopsy was performed at initial diagnosis. As per investigator assessment, 68:5% of patients (*n* = 317) had a diagnosis according to the WHO criteria from 2008. It was then up to the treating physician to report the biopsies in detail on a data sheet in the eCRF. For 343 patients, a detailed description was provided, and 314 patients of those were reported to have evaluable biopsies. Furthermore, 59.7% of patients (*n* = 277) had not undergone BM examination during the year before enrollment into the study; 187 patients were reported to have evaluable BM biopsies. Intriguingly, and perhaps counterintuitively, dry taps (*punctio sicca*) were not frequent, and physicians reported evaluable BM aspirations in as many as 86.7% of cases. In total, 67.5% of BM biopsies were reported to be hypercellular or strongly hypercellular, 14.1% were normocellular, and 17.2% were hypocellular. Histologic staining was reported in only 136 cases, with Grade 0–1, Grade 2, or Grade 3 MF (scale of 0–3) diagnosed in 28.0%, 45.6%, and 26.5% of cases, respectively; 43.9% of patients had 0% blasts in their BM biopsies, and only six cases showed blast counts above 5% (up to 14%).

At study entry of patients, physicians were asked if they had performed a molecular analysis of the patients before study start. A molecular analysis, which was not mandatory, was done in 83% (*n* = 385) of the patients. It was then at the discretion of the treating physician to report the results of the mutational analysis in detail on a data sheet in the eCRF. For 295 patients, a detailed description on their molecular status was provided.

As stated above, the study protocol did not provide mandatory examinations, so this may reflect the real-world behavior of German physicians treating MF patients. Only 151 (32.5%) patients had at least one molecular examination performed as a possible examination in the study (up to three in some patients), and results were reported from 140 patients (30.2%), with 110 of these patients harboring the *JAK2V617F* mutation, three patients with *MPL W515* mutations, and 22 patients harboring other mutations (three patients with *CALR* mutations; 78 patients (16.8%) had cytogenetic results available from the last year before baseline. The low numbers of patients with results and with *CALR* mutations reflect the low prevalence of these tests at the time of trial initiation in 2012.

### Efficacy of RUX treatment

The median RUX dose administered was 23.77 mg/day. Patients were started on a median dose of 30 mg/day (equivalent to 15 mg twice daily [BID]), not differing between the three subentities. 39.5% and 25.4% of patients received 30 mg/day or 40 mg/day, respectively; however, 35.1% of patients were dosed with 20 mg/day or less, and 20.8% of patients received 10 mg/day or less. There were no relevant differences between PMF, PPV-MF, or PET-MF patients regarding average, start, or last doses of RUX. Median treatment duration was 1.5 years, with a slightly shorter duration in patients with PMF and PET-MF than in PPV-MF patients. As expected, patients who dropped out showed a lower median average dose and a lower median treatment duration than patients who had completed the trial (20.8 mg/day vs 25.7 mg/day, *p* = 0.0549; 0.7 years vs 3.0 years, *p* ≤ 0.0001).

67% of patients remained on RUX continuously while 33% of patients interrupted RUX between one and nine times during the trial. The most frequent reasons for RUX interruption were AEs, particularly thrombocytopenia. Interestingly, patients who dropped out had not interrupted RUX significantly more frequently than those completing the trial. On the other hand, only 29.5% of patients received the same dose throughout the trial, while fewer than 20% each were subjected to at least one or two dose adjustments, respectively, with the rest experiencing up to 18 dose adjustments. Most dose adjustments were due to AEs, particularly thrombocytopenia. Kidney dysfunction was not reported as a reason for dose adjustment at all, and liver dysfunction was cited as the reason for dose adjustment once.

Only 31.5% of patients completed the full 36 months on trial, while 54.3% of patients dropped out before reaching this time point. At the time of analysis, 14.2% of patients were still receiving treatment. Reasons for early drop out from the trial can be found in Table 4.

**Table 4:** Study completion status at data-cutoff and reasons for early study discontinuation (*n* = 464)

	No diagnosis (*N* = 3) *n* (%)	PMF (*N* = 310) *n* (%)	PPV-MF (*N* = 96) *n* (%)	PET-MF (*N* = 55) *n* (%)	Total (*N* = 464) *n* (%)
Study completed					
Yes (completer)	0	82 (26.5)	45 (46.9)	19 (34.5)	146 (31.5)
No (drop out)	2 (66.7)	181 (58.4)	40 (41.7)	29 (52.7)	252 (54.3)
On treatment	1 (33.3)	47 (15.2)	11 (11.5)	7 (12.7)	66 (14.2)
Reason for premature termination of documentation					
Poor compliance	0	5 (1.6)	1 (1.0)	2 (3.6)	8 (1.7)
Lost to follow-up	1 (33.3)	21 (6.8)	3 (3.1)	3 (5.5)	28 (6.0)
Therapy response	0	16 (5.2)	2 (2.1)	4 (7.3)	22 (4.7)
Progression of primary disease	0	18 (5.8)	2 (2.1)	5 (9.1)	25 (5.4)
Change to AML	0	7 (2.3)	0	0	7 (1.5)
AE	0	41 (13.2)	13 (13.5)	4 (7.3)	58 (12.5)
Deterioration of general health	0	24 (7.7)	4 (4.2)	5 (9.1)	33 (7.1)
Death	0	60 (19.4)	13 (13.5)	7 (12.7)	80 (17.2)
Infection	0	1 (0.3)	0	0	1 (0.2)
Other	1 (33.3)	33 (10.6)	8 (8.3)	9 (16.4)	51 (11.0)

Abbreviations: *AE* adverse event, *AML* acute myeloid leukemia, *PET-MF* post-essential thrombocythemia myelofibrosis, PMF primary myelofibrosis, *PPV-MF* post polycythemia vera myelofibrosis

Among patients who dropped out, the combined fraction of high-risk and int-2–risk patients was over-represented (68.4% of drop-out patients); however, median study duration was comparable between the IPSS risk groups: (low: 17.5 months, int-1: 17.9 months, int-2: 17.7 months, high: 17.9 months; total: 17.8 months).

### Spleen response during RUX treatment

**Fig. 2:**
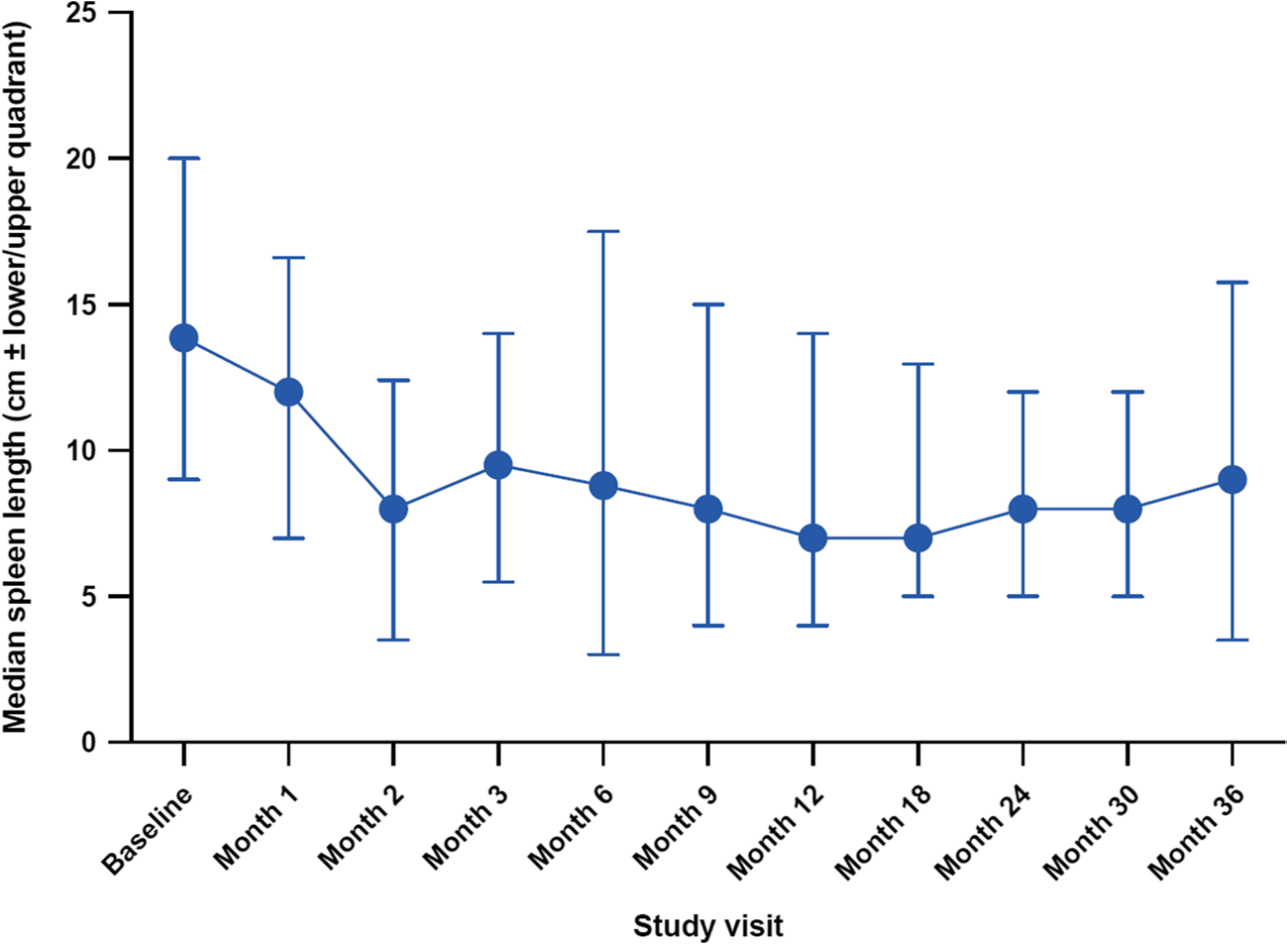
Spleen length below costal margin by palpation during the course of the trial. Median with lower and upper quadrants (*n* = 464)

Figure 2 shows change in spleen length by palpation over the course of the trial. The results show that median spleen length was markedly and rapidly reduced from 13.9 cm BCM at baseline to 8.0 cm after 2 months of therapy (median change of –4.0 cm vs baseline), and spleen length reductions remained stable over the course of the trial.

The extent of spleen length reduction among the three disease subentities (PMF, PPV-MF, and PET-MF) was similar, with the largest reduction observed in patients with PPV-MF and the smallest in patients with PET-MF, but the effect was dose dependent, with a more pronounced reduction observed with 40 mg/day RUX compared with 20 mg/day or 10 mg/day.

Spleen assessment by abdominal ultrasound was performed in 75.7% of cases at baseline. While spleen volume was only rarely reported, spleen length by ultrasound was frequently reported, with a median length of 18.5 cm. Spleen length by ultrasound after 2 months of RUX therapy was reported as 16.2 cm (median change of –2.0 cm vs baseline). The extent of spleen length reduction by ultrasound was again highest in the PPV-MF group and lowest in the PET-MF group, and dose dependent, with the most pronounced dose reduction seen with 40 mg/day RUX.

**Fig. 3:**
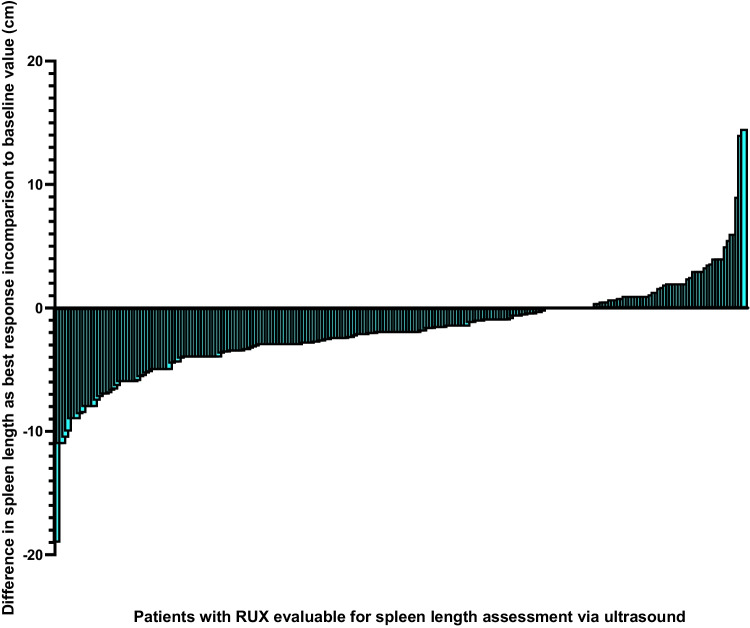
Differences in spleen length measurement from baseline to last post-baseline visit by ultrasound**.** Abbreviations: *RUX* ruxolitinib. Mean duration from baseline to the last assessment of spleen size by ultrasound was 18 months. Median time to last spleen measurement by ultrasound was 16.6 months

**Fig. 4:**
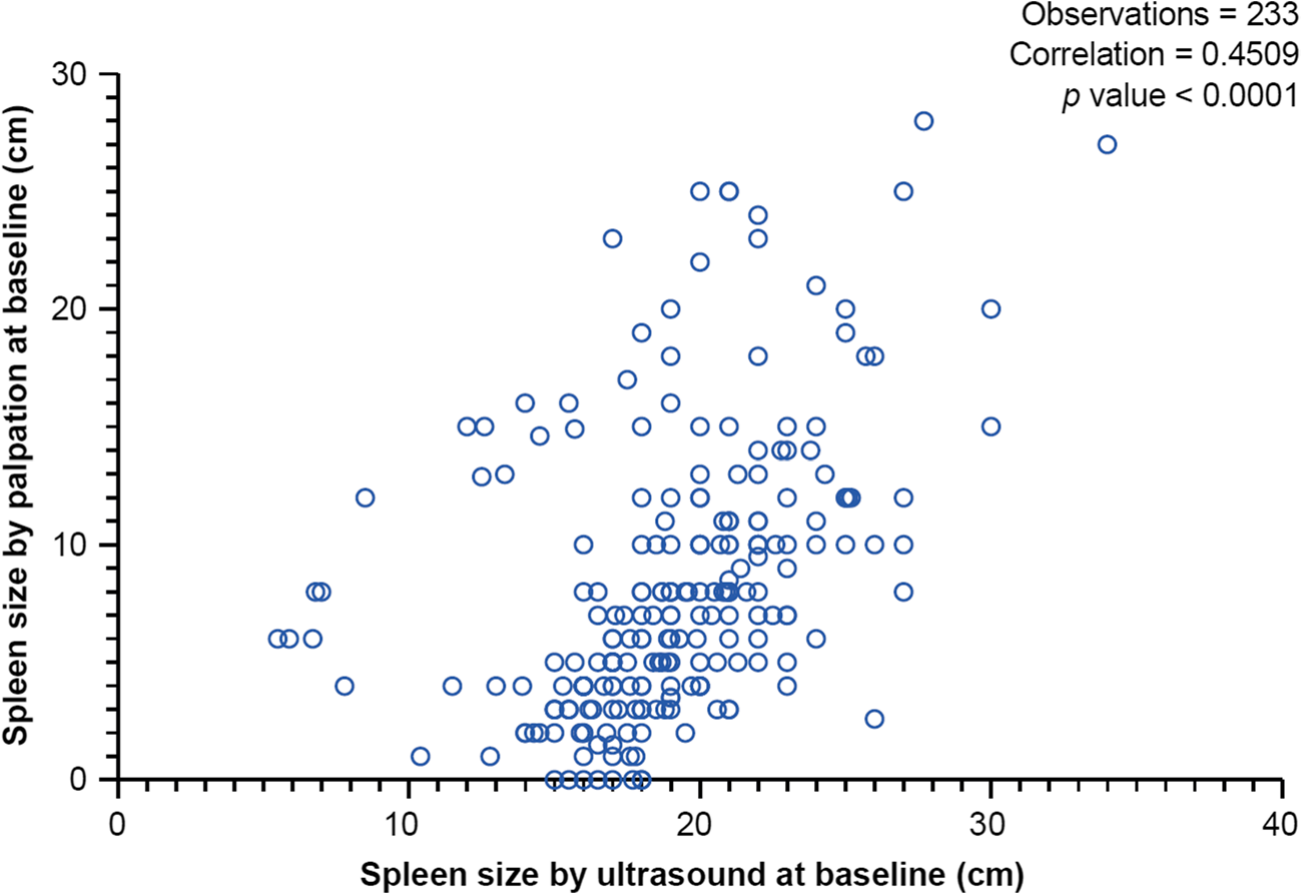
Pearson correlation between spleen size measured by palpation vs in ultrasound (*n* = 233). Identical values have been deleted. Spleen size by palpation is analyzed as values below costal margin

Interestingly, correlation between spleen size measured by palpation (values BCM) vs spleen length by ultrasound was accurate (Fig. 3, Fig. 4), although all spleen size values measured by ultrasound were supposed to be at least 14 cm larger (as a normal spleen is usually not palpable BCM, 14 cm was estimated to be the smallest spleen size to be palpable). This might show a reporting bias, as some of the investigators might have reported not only the palpable part of the spleen in centimeters BCM, but the unpalpable part as well. MRI or CT assessment was only performed in 7.4% of patients, consistent with current practice of not using these techniques for routine work-up of MF patients.

### General condition and symptom burden during RUX treatment

ECOG performance status of patients showed a trend towards improvement after RUX treatment (16.5% of patients with ECOG 2–4 at baseline vs 12.7% at Month 36), with the proportion of patients with no restrictions (ECOG 0) increasing over time (29.9% at baseline vs 43.7% at Month 36). In 29.0% of patients, the ECOG performance status decreased, while it tended to be increased in 18.8% of patients at Month 36, as compared with baseline (*p* = 0.0623, sign test). Furthermore, there was a significant improvement in symptom burden during RUX treatment, as reported by both physicians and patients.

When assessed by physicians, overall constitutional symptoms were present in 66.2% of patients at baseline, but only in 37.2% patients (*p* < 0.0001) at Month 1, 31.3% (*p* < 0.0001) at Month 2, and 20.3% (*p* < 0.0001) at Month12, remaining stable afterwards until the end of follow-up at Month 36. There were no major differences between the three disease subentities. Similarly, weakness (48.5% at baseline vs 13.4% at Month 36; *p* < 0.0001), sweating (26.1% vs 7.0%; *p* < 0.0001), fever (2.2% vs 0.6%; *p* < 0.0117), pruritus (13.8% vs 4.5%; *p* < 0.0001), bone pain (9.1% vs 4.5% *p* < 0.0001), and weight loss (25.4% vs 2.5%; *p* < 0.0001) decreased during RUX treatment.

**Fig. 5:**
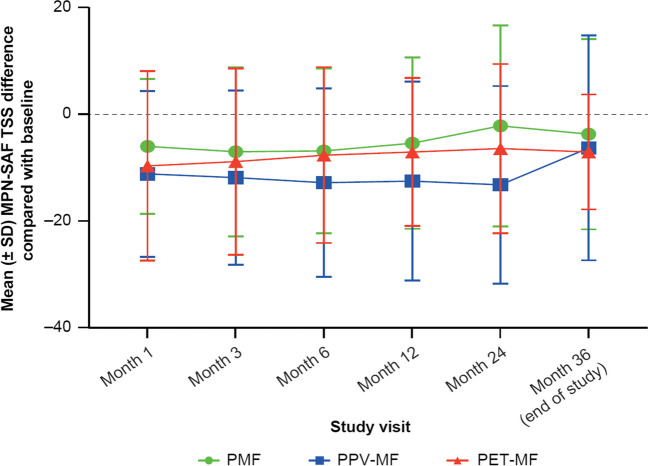
MPN-SAF TSS changes over time in comparison to baseline. MPN-SAF TSS difference is shown in comparison to baseline for total patient cohort and divided by subentity *n* = 464). Abbreviations: *MPN-SAF* Myeloproliferative Neoplasm Symptom Assessment Form, *TSS* total symptom score, *PMF* primary myelofibrosis, *PPV-MF* post-polycythemia vera myelofibrosis, *PET-MF* post-essential thrombocythemia myelofibrosis

Also, upon patient self-assessment via the internationally accepted MPN-SAF questionnaire, there was a significant and relevant reduction in the number of symptoms reported, as measured by the TSS: TSS improved by 5.2 points at Month 1 and was maximally reduced by 7.4 points at Month 3, after which the reduction lessened but remained significantly improved. In total, quality of life, as reported by patients, improved significantly (*p* = 0.0308). At the end of the study, 41.2% of patients reported an at least five-point improvement in TSS, and 26.8% of patients reported an at least 50% improvement in TSS vs baseline (Fig. 5).

### Overall survival and progression

**Fig. 6:**
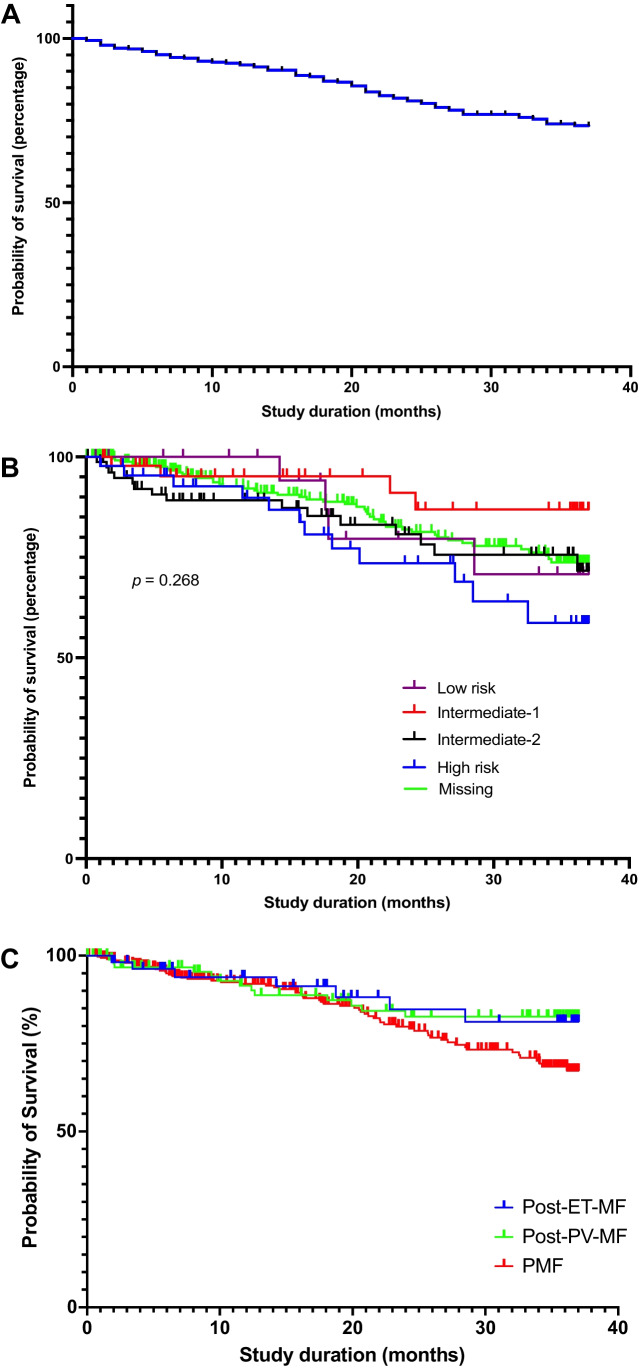
Overall survival analysis in JAKoMo patients (Kaplan–Meier estimator). (**A**) Survival analysis for all patients; (**B**) Survival analysis for patients according to their IPSS risk score at baseline. *p* = 0.268 by Log-rank (Mantel–Cox) test (**C**) Survival analysis for patients according to their myelofibrosis subtype

24-month overall survival after RUX initiation was 81.2% for the entire cohort. Survival was dependent on IPSS scores, with survival in patients with low-, int-1–risk, int-2–risk, and high-risk scores being 79.6%, 91.1%, 80.7%, and 73.5%, respectively. Potential inconsistencies in these data are most likely caused by the large number of patients with missing IPSS data (270 out of 464 patients). Figure 6 shows the Kaplan–Meier plots of survival of the whole cohort and of the IPSS-stratified cohorts.

### Progression/death

80 (17.2%) patients died during the study, 25 (5.4%) patients terminated the study due to progression of primary disease, and seven (1.5%) patients terminated the study because of transformation to acute myeloid leukemia (AML).

### Safety and effects on lab parameters

#### AEs

86.9% of all patients experienced at least one AE (Table 5), the most common being anemia (42.0%) and thrombocytopenia (29.1%), and 51.3% of patients experienced at least one serious AE (SAE), most commonly anemia.

**Table 5:** AEs independent of relationship to treatment and proportion of SAEs (*n* = 464)

System organ class	AE term	AEs all grades (% of patients)	SAEs (% of patients)
Blood and lymphatic system disorders		51.9	11.4
	Anemia	42.0	11.9
	Thrombocytopenia	29.1	5.0
	Splenomegaly	8.8	0.9
Cardiac disorders		12.9	9.9
Gastrointestinal disorders		28.9	10.6
	Abdominal pain	5.4	1.3
	Diarrhea	9.5	2.4
	Nausea	7.1	0.9
General disorders and administration site conditions		42.7	17.5
	Asthenia	12.3	1.9
	Fatigue	12.7	0.6
	General physical health deterioration	8.2	5.6
	Pyrexia	6.9	3.4
Infections and infestations		37.1	17.9
	Pneumonia	7.3	5.4
	Urinary tract infection	6.9	3.2
Injury, poisoning, and procedural complications		11.4	6.7
Investigations		33.8	13.8
	Hemoglobin decreased	11.2	6.3
Metabolism and nutrition disorders		14.9	4.1
Musculoskeletal and connective tissue disorders		20.3	4.7
	Bone pain	5.0	0.2
Neoplasms (benign, malignant, and unspecified, including cysts and polyps)		17.5	13.4
	Myelofibrosis	6.7	4.7
Nervous system disorders		22.4	6.5
	Dizziness	9.5	0.9
	Headache	6.5	0.4
Psychiatric disorders		7.1	1.1
Renal and urinary disorders		9.3	5.4
Respiratory, thoracic, and mediastinal disorders		22.6	9.7
	Dyspnea	7.5	2.8
	Epistaxis	5.8	1.1
Skin and subcutaneous tissue disorders		18.8	1.3
	Night sweats	5.8	–
	Pruritus	6.5	0.2
Vascular disorders		12.9	6.0

Only AEs that occurred in ≥ 5% of patients are shown here

Abbreviations: *AE* adverse event, *SAE* serious adverse event

#### SAEs

SAEs were reported in 51.3% of patients. SAEs that occurred in at least 5% of patients included anemia (11.9%), thrombocytopenia (5.0%), general physical health deterioration (5.6%), and pneumonia (5.4%). For SAEs occurring in more than 1% of patients, please see Table 6.

**Table 6:** SAEs independent of relationship to treatment (*n* = 464)

System organ class	SAE term	SAEs (% of patients)
Blood and lymphatic system disorders		11.4
	Anemia	11.9
	Thrombocytopenia	5.0
	Leukocytosis	1.1
Cardiac disorders		9.9
	Atrial fibrillation	2.8
	Cardiac failure	3.2
	Coronary artery disease	1.3
Gastrointestinal disorders		10.6
	Abdominal pain	1.3
	Diarrhea	2.4
	Ascites	1.3
	Vomiting	1.3
General disorders and administration site conditions		17.5
	Asthenia	1.9
	Death	4.3
	General physical health deterioration	5.6
	Multiple organ dysfunction syndrome	1.5
	Edema peripheral	1.9
	Pyrexia	3.4
Infections and infestations		17.9
	Herpes zoster	1.1
	Infection	1.1
	Peritonitis	1.3
	Pneumonia	5.4
	Sepsis	3.0
	Urinary tract infection	3.2
	Urosepsis	1.3
Injury, poisoning, and procedural complications		6.7
	Fall	3.4
Investigations		13.8
	LDH increased	6.3
	CRP increased	2.4
Neoplasms (benign, malignant, and unspecified, including cysts and polyps)		13.4
	Myelofibrosis	4.7
	AML	1.3
	Transformation to acute leukemia	1.3
Renal and urinary disorders		5.4
	Acute kidney injury	1.7
	Renal failure	1.5
Respiratory, thoracic, and mediastinal disorders		9.7
	Dyspnea	2.8
	Epistaxis	1.1
	Pleural effusion	1.5
Vascular disorders		6.0
	Pulmonary embolism	2.4
	Deep vein thrombosis	1.3
	Hypertensive crisis	1.1

Only events occurring in ≥ 1% of patients are shown in this table. Abbreviations: *AML* acute myeloid leukemia; *CRP* C-reactive protein, *LDH* lactate dehydrogenase; *SAE* serious adverse event

#### Hematologic toxicity/parameters

The most common adverse hematologic events were anemia (all grades, 42.0%) and thrombocytopenia (all grades, 29.1%; Table 5); however, only 1.5% (*n* = 7) and 1.9% (*n* = 9) of patients, respectively, discontinued treatment, indicating that these AEs were manageable in most patients.

Median hemoglobin levels decreased initially upon RUX treatment but subsequently stabilized or even returned to their initial values (from 11.1 g/dL at baseline to 9.8 g/dL at Month 2 and 11.0 g/dL at the end of the study; *p* < 0.0001 for all visits, Wilcoxon rank-sum test). However, as expected, baseline hemoglobin levels differed between the three MF subentities, and, while median hemoglobin eventually recovered in patients with PMF, it remained significantly decreased in patients with PET-MF and PPV-MF at the end of the study.

Median WBC count decreased during RUX treatment from 13.3 × 10^9^/L at baseline to 8.3 × 10^9^/L at the end of the study (*p* < 0.0001). While the WBC count was initially highest in patients with PPV-MF, this decrease was seen across all three MF subentities and, at the end of the study, the median WBC count was around 8 × 10^9^/L in all MF subentities.

During RUX treatment, the median number of platelets initially decreased from 287 × 10^9^/L at baseline to 181 × 10^9^/L at 2 months (*p* < 0.0001) but increased afterwards to 209 × 10^9^/L at the end of the study.

#### Non-hematologic toxicity

The most common non-hematologic AEs (occurring in ≥ 5% of patients) included nausea, diarrhea, pyrexia, fatigue, and asthenia. Atrial fibrillation (AF) occurred in 3.9% of patients (patients with concomitant AF were excluded from the COMFORT trials).

Elevated alkaline phosphatase (AP) levels have been described in Philadelphia chromosome–negative MPNs, and *JAK2V617F* induces leukocyte AP; therefore, we investigated whether RUX treatment changed AP levels in our cohort overall. Indeed, median AP levels decreased from 99.0 U/L at baseline to 67.0 U/L at the end of the study (*p* = 0.0068).

There were no relevant changes in bilirubin, aspartate aminotransferase, alanine aminotransferase, gamma-glutamyl transferase, glutamate dehydrogenase, serum protein, or lipase levels during RUX treatment. Also, urea, sodium, potassium, and chloride levels were not elevated at baseline and did not change relevantly during RUX treatment. Median creatinine slightly increased over time, with the increase being most severe in patients with PET-MF and PPV-MF (*p* < 0.0001).

#### Infections

37.1% of patients developed infections during the trial, with 8.4%, 14.4%, and 13.4% of patients experiencing mild, moderate, and severe infections, respectively. 17.9% of patients experienced infection-associated SAEs. Herpes zoster infection occurred in 4.7% of patients. There were no cases of viral hepatitis (new diagnosis or reactivation) or tuberculosis reactivation. One single case of progressive multifocal leukoencephalopathy was reported.

## Discussion

Here, we report on the largest German cohort of patients with MF treated according to the approved label with RUX almost exclusively by hematologists/oncologists in the outpatient office setting and, thus, reflective of the true, real-world scenario of RUX treatment as per the SmPC. This interim analysis focusses on 464 patients with PMF, PPV-MF, or PET-MF not previously treated with RUX or any other JAKi (Arm A).

There were no further restrictions regarding comorbidities, dynamic (D)IPSS risk, or spleen size. Patients with low-risk disease were included independent of their blood counts. Despite the liberal inclusion criteria, median spleen length at baseline (14 cm BCM) was comparable to the JUMP (13.3 cm) and COMFORT-II (14 cm) trials [10, 13]. Despite the fact that BM biopsy results were only available for a fraction of patients, almost all of them (93%) had evaluable BM biopsies, and, perhaps unexpectedly, dry taps were infrequent, with evaluable BM aspirations reported in 86.7% of patients. This is encouraging and suggests that futile aspirations may not be as frequent as previously reported.

One rather surprising finding was the high percentage (58%) of missing data for IPSS (or DIPSS) scoring, suggesting that physicians involved in this trial did not employ (D)IPSS scoring when deciding to commence RUX treatment. This contrasts current guidelines by the German Society of Hematology and Medical Oncology [17], which recommend (D)IPSS scoring in order to decide on therapy, but is in accordance with the European Medicines Agency label, allowing for RUX treatment in patients with MF-associated symptoms and/or splenomegaly regardless of (D)IPSS score; however, as expected, this high rate of missing data was not seen in the JUMP (17.4%) or two COMFORT trials (0% missing in the RUX arms), since the risk score was one of the eligibility criteria in these trials. Missing (D)IPSS scores most likely contributed to inclusion of a different pt population in our JAKoMo trial (only 59% int-2–risk or high-risk patients) than in the COMFORT-II trial (100% of patients being int-2 risk and high risk, as per inclusion criteria). The sizable fraction of low-risk to int-1–risk JAKoMo patients (around 40%) more likely reflects the current routine regarding RUX treatment of MF patients in Germany; however, as only about 60% of patients participating in the JAKoMo trial had either documented or retrospectively calculated IPSS scores, the true percentages of patients in each risk category may differ slightly from what we report here. The lack of retrospectively assessable IPSS scores was mainly due to missing blast counts, which might reflect the low prevalence of this parameter during routine care of MF patients in Germany. As this was a real world analysis, study protocol did not provide specific instructions what to do at which time point at which parameters had to be collected. Furthermore, monitoring was limited.

Interestingly, the JAKoMo study cohort showed signs of both earlier disease (few dry taps, lower IPSS scores) and, concomitantly, more advanced disease, with around half of the patients in the JAKoMo trial (48.5%) having been transfusion dependent at baseline, almost twice as much as in the JUMP trial (25.9%) [13]. The JAKoMo pt cohort was also older (median age 73 years vs 67 years in both the COMFORT-II and JUMP trials [10, 11, 13]). Accordingly, our cohort contained both a smaller fraction of ECOG 0 patients and a higher fraction of ECOG 2–3 patients compared with COMFORT-II. This suggests that IPSS is not as suitable as more recent risk scores such as DIPSS and the Mutation-Enhanced International Prognostic Scoring System 70, which apply more leverage to anemia and transfusion dependence.

Median treatment duration in patients participating in the JAKoMo trial was 1.5 years, a little longer than in the JUMP trial. Primary reasons for RUX discontinuation were also similar in both trials, particularly thrombocytopenia and disease progression. Importantly, kidney and liver dysfunction were almost never stated as reasons for dose adjustments in our trial, suggesting that this was not deemed a significant clinical problem.

Median spleen length was markedly and rapidly reduced from 13.9 cm BCM at baseline to 8.0 cm after 2 months of therapy (reduction of 42%); this was comparable to efficacy reported in the JUMP trial (40%) and slightly lower than that reported in the COMFORT-II (56%) trial. Comparison of spleen length by palpation and by ultrasound suggested that spleen length by palpation may be overestimated; however, despite the overall overestimation by palpation, intraindividual correlation between both measures in our patients was very accurate, leading us to conclude that overestimation by palpation does not vary between several visits. As spleen sizes via palpation correlated well with ultrasound length measurements, this suggests that palpation is a cost-effective and simple alternative in the routine setting, but it might overestimate actual spleen size.

Since the beginning of RUX clinical development, clinical trials in MF have included spleen size and symptom reductions as clinical endpoints. Although these endpoints translate into benefits for patients by providing better quality of life, a landmark trial [18] demonstrated that treatment goals and the perception of priorities can differ considerably between physicians and patients. This discrepancy was subsequently confirmed by a European study [19, 20]. Physicians perceived symptom improvement and a decrease in spleen size as being important to patients, while patients were more likely to emphasize the importance of a survival benefit and a modification of the natural course of the disease. Although the data are subject to debate, a pooled long-term analysis of both COMFORT trials showed improved overall survival with RUX treatment [12] along with reduction of splenomegaly and, in a fraction of patients, a decrease in BM fibrosis [21]. Future drugs will be compared to RUX in these regards.

In the real-world setting of our study, RUX was fairly well tolerated, revealing no new safety signals. As described, the most frequent AEs included anemia in 31% and thrombocytopenia in 27% of patients. Anemia-related SAEs occurred in 13% of patients in this population (in comparison to 4.5% of anemia SAEs in the JUMP trial [22] and 5% of anemia SAEs in the COMFORT-II trial), while almost half of JAKoMo trial patients were initially transfusion dependent at baseline, so this seems to be comparable to both the JUMP and COMFORT trials. At the time of analysis (median study duration of 13.1 months), 42% of patients had dropped out of the study, which is comparable to the other clinical trials, as recently summarized [23].

In addition to the points addressed above, limitations to this analysis include the observatory character of the trial with limited on-site monitoring of the data, and this may also explain the missing values, particularly regarding IPSS scoring and molecular genetics assessments.

## Conclusions

In conclusion, this large analysis of RUX treatment in therapy-naïve MF patients confirms feasibility in the real-world setting and demonstrates efficacy and tolerability comparable to those observed in the more selected JUMP and COMFORT-II drug approval trial populations.

## Data Availability

The datasets generated and/or analyzed during the current study are not publicly available but are available from the corresponding author on reasonable request and with permission of the steering committee.
